# Human colorectal cancer cells induce vascular smooth muscle cell apoptosis in an exocrine manner

**DOI:** 10.18632/oncotarget.18893

**Published:** 2017-06-27

**Authors:** Wei-Wei Li, Hai-Yue Wang, Xi Nie, Ya-Bin Liu, Mei Han, Bing-Hui Li

**Affiliations:** ^1^ Department of Biochemistry and Molecular Biology, College of Basic Medicine, Key Laboratory of Medical Biotechnology of Hebei Province, Shijiazhuang 050017, P. R. China; ^2^ Department of Surgery, Fourth Affiliated Hospital, Hebei Medical University, Shijiazhuang 050017, P. R. China

**Keywords:** colorectal cancer (CRC), vascular smooth muscle cells (VSMCs), apoptosis, tumor microenvironment, tumor angiogenesis

## Abstract

Tumor vessels often lack the smooth muscle layer, and the instability is conducive to tumor invasion and metastasis. The effect of tumor microenvironment on vascular smooth muscle cells needs to be explored. In the present study, we examined the density of the tumor vessels in human colorectal cancer tissues, and used the tumor conditioned medium of human colorectal cancer HT29 cells to mimic the tumor microenvironment. We showed that the vessel density in colorectal cancer tissues increased, which displayed a decreased expression of smooth muscle α-actin, a specific marker of vascular smooth muscle cells and an attenuated or a discontinuous layer of vascular smooth muscle cells compared with the matched normal tissues. We also showed that the tumor conditioned medium decreased the cell viability, and induced the apoptosis in vascular smooth muscle cells in a concentration-dependent manner. The expression of pro-Caspase-3 was down-regulated, accompanied by increasing of cleaved-Caspase-3 in the cells treated with the tumor conditioned medium, suggesting that Caspase-3 was activated. Moreover, the expression of Bax was increased, and the ratio of Bcl-2/Bax was decreased under the same conditions. Furthermore, the treatment with the tumor conditioned medium resulted in loss of mitochondrial membrane potential in vascular smooth muscle cells. These findings suggest that HT29 cells induce apoptosis of vascular smooth muscle cells in an exocrine manner, associated with activating caspase-3 via mitochondrial apoptotic pathway. This may be one of the mechanisms underlying tumor vascular structural abnormalities.

## INTRODUCTION

Colorectal cancer (CRC) is the third most common malignancy and the fourth leading cause of cancer-related deaths worldwide. Despite improvements in diagnosis and treatment, more than 50% of patients diagnosed with CRC will eventually die from their disease. Approximately 90% of CRC-related mortalities are caused by direct or indirect effects of metastatic dissemination [1, 2].

Angiogenesis is enssential in tumor growth and metastasis, and is tightly regulated by pro- and anti-angiogenic factors produced by both malignant cells and nonmalignant cells [3, 4]. Vascular endothelial growth factor (VEGF)/VEGF-A and platelet derived growth factor (PDGF) are the predominant proangiogenic factors that are involved in vascular cell activation [5, 6]. Different from the normal vasculature, the tumor vessels are primitive, lack a continuous smooth muscle layer, and often are comprised of an endothelial layer and a connective tissue alone, resulting in a leaky vascular system, which promotes metastasis by facilitating the movement of tumor cells into the blood stream [7, 8]. However, the mechanism underlying lack of a muscular coat in tumor vessels is unclear.

The present study was designed to investigate the effect of the tumor microenvironment on vascular smooth muscle cells (VSMCs). We used the tumor conditioned medium (TCM) from human CRC cell line HT29 cells to mimic tumor microenvironment, and examined its effect on the proliferation and apoptosis of VSMCs *in vitro.* Our findings suggested that human CRC cells induced VSMC apoptosis in an exocrine manner throuth activating the caspase-3 via mitochondrial apoptotic pathway.

## RESULTS

### The level of SMA expression is declined accompanied with increased vessel density in colorectal cancer tissues

**Figure 1:**
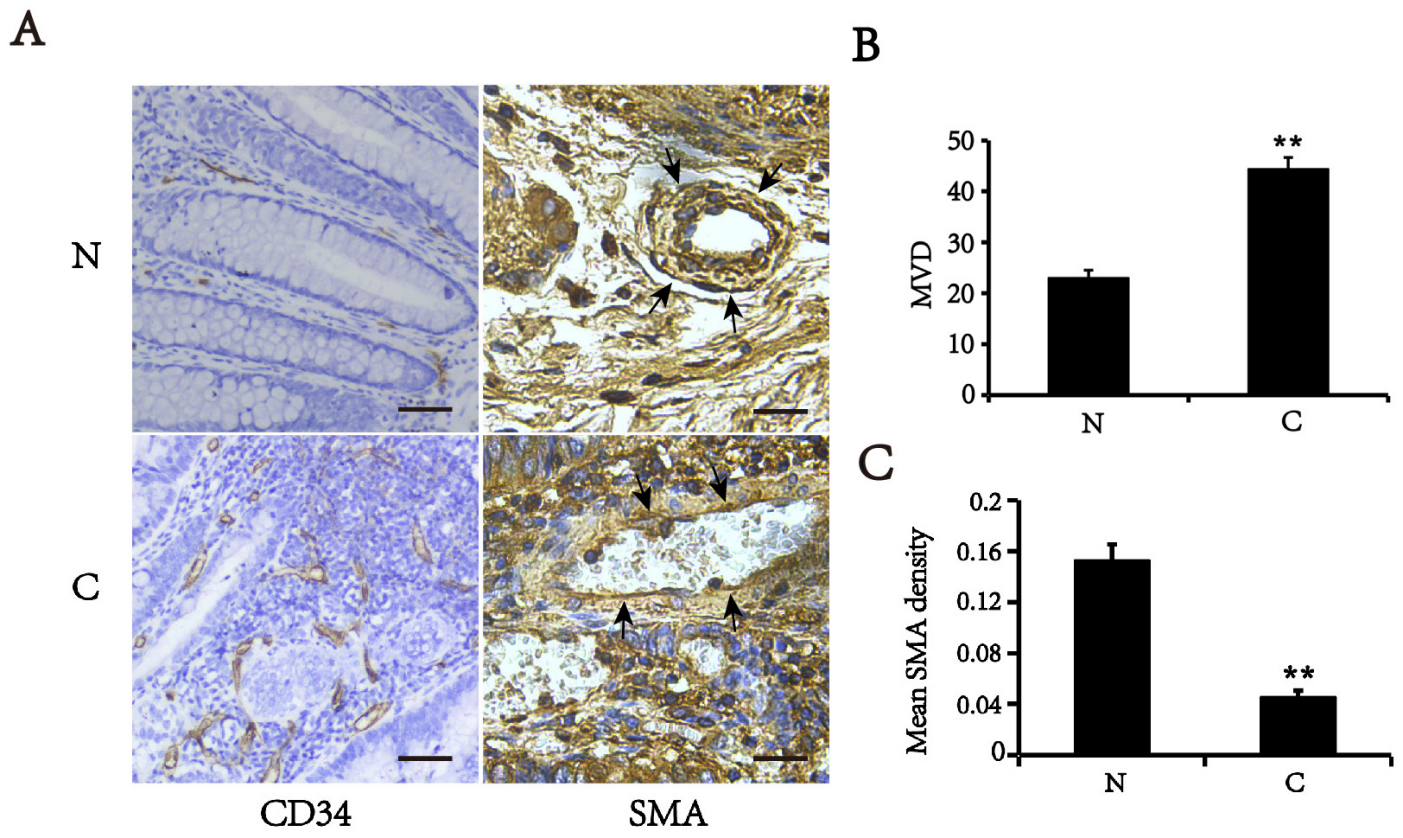
Expression of SMA and CD34 in colorectal cancer (C) and matched normal tissues (N) **(A)** Typical images of immunohistochemical staining of SMA and CD34 (Area pointed by arrows). Bars, 50 μm. **(B)** MVD counts in colorectal cancer and corresponding normal tissues. **(C)** Mean optical density of SMA in vascular walls. All of values are mean ± SEM from three independent experiments (n=5). **P < 0.01 versus matched normal tissues.

Tumor blood vessels display poor coverage by smooth muscle cells [8]. To validate these findings, we first detected the expression of CD34 (endothelial cell marker) and smooth muscle α-actin (SMA), a specific marker of VSMCs, in CRC and their matched adjacent normal tissues by immunohistochemistry (Figure 1). We showed that the microvascular density (MVD) was increased in colorectal cancer tissues, compared with matched normal tissues (*p*<0.01). However, the level of SMA expression in the tumor vessels was significantly lower than that in matched normal tissues. The vessels in normal tissues displayed a constant high density of VSMCs that was identified by the SMA positive staining. Conversely, the vessels in CRC tissues had an attenuated or discontinuous density of VSMCs. These findings suggest that the colorectal cancer vessels have either only a slight or lacking muscular coat.

### TCM treatment results in a decreased VSMC viability

**Figure 2:**
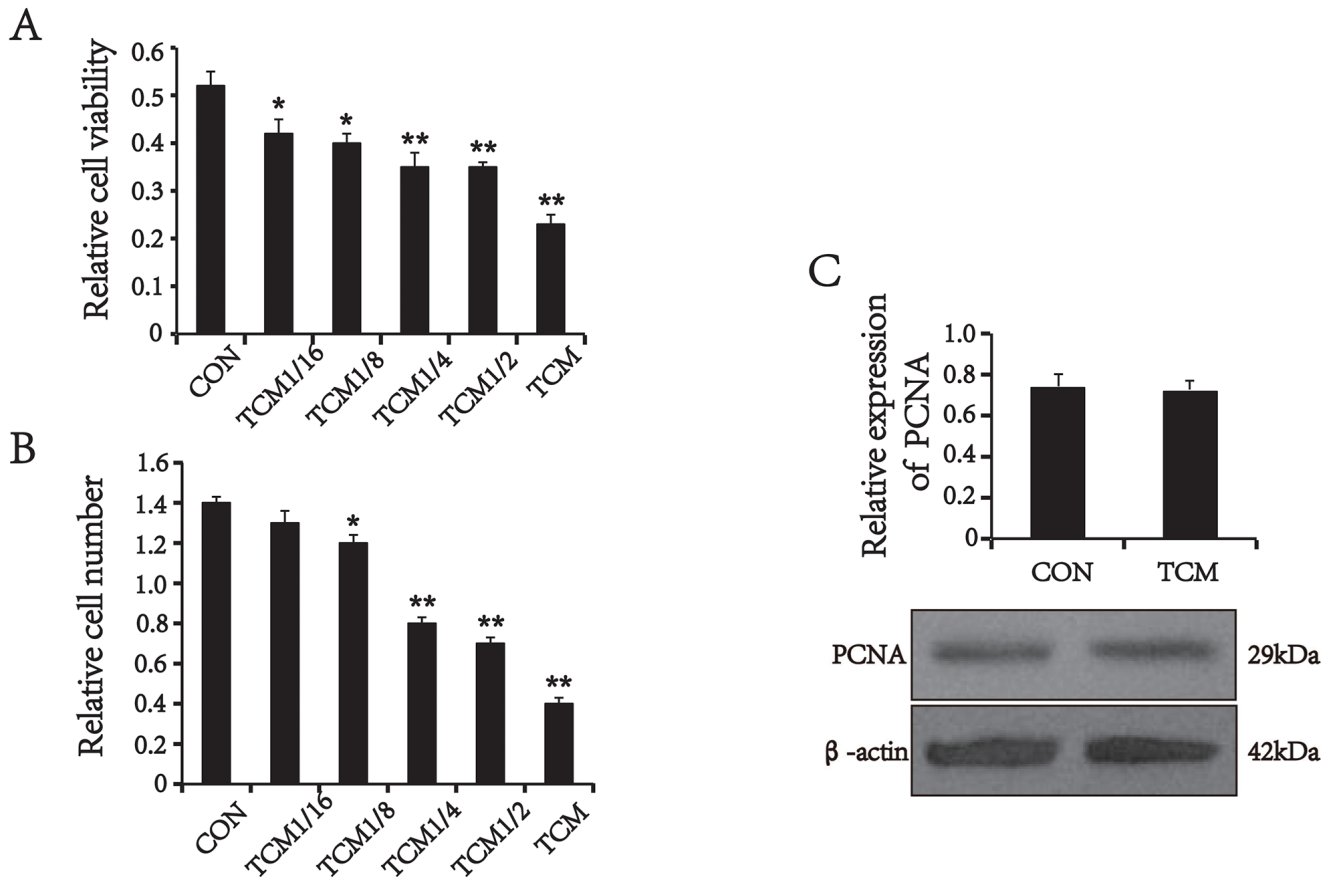
The effect of TCM on VSMC proliferation and the expression of PCNA **(A)** MTT assays (n=5). **(B)** Cell counts (n=5). **(C)** Western blot analysis and quantification for PCNA (n=3). Values are mean ± SEM from three independent experiments. *P < 0.05, **P < 0.01 versus the CON.

To investigate the mechanism underlying decreasing of SMA expression in colorectal cancer vessels, we first examined the effects of the tumor cells on VSMCs. VSMCs were treated with different concentrations of the TCM from human CRC cell line HT29 cells for 24 h. The viability of VSMCs was determined by the 3-(4,5-dimethylthiazol-2-yl)-2, 5-diphenyltetrazolium bromide (MTT) assay. The results showed that TCM reduced the viability of VSMCs in a concentration-dependent manner (Figure 2A). This finding was validated by cell counts (Figure 2B). Next, proliferating cell nuclear antigen (PCNA), a marker of cell proliferation, was detected by Western blot analysis. Unexpectedly, the level of PCNA expression in TCM-treated VSMCs was not changed compared with the control (*P*>0.05) (Figure 2C), suggesting that TCM don’t inhibit the proliferating activity of VSMCs. Thus, we speculated that the decreased cell number may be associated with apoptosis.

### TCM induces VSMC death via mitochondrial apoptotic pathway

**Figure 3:**
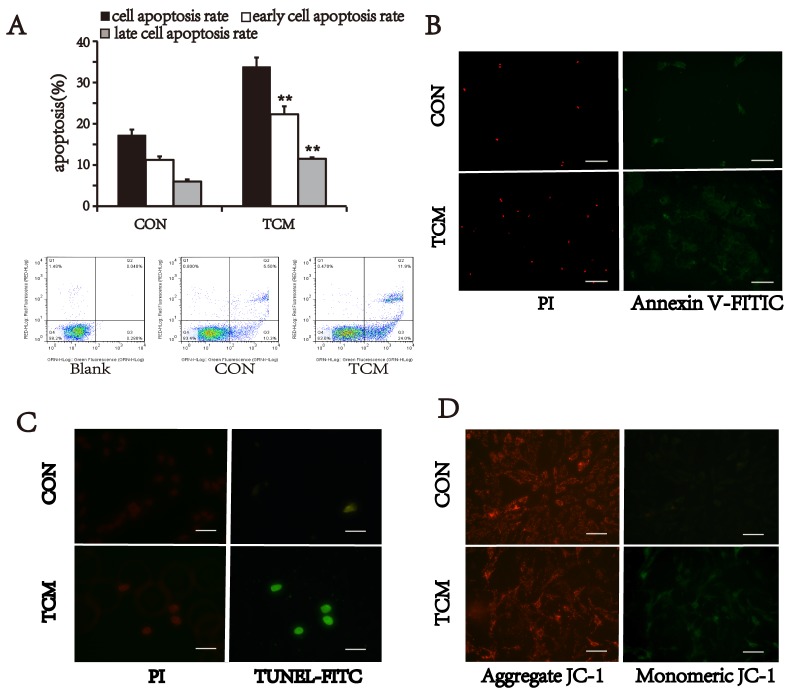
Effect of TCM on VSMC apoptosis **(A)** VSMC apoptosis detected by flow cytomety with Annexin V-FITC/PI staining. Bar graphs show the means ± SEM from three independent experiments (n=3). **P < 0.01 versus the CON. **(B)** Representative images of Annexin V-FITC/PI staining. **(C)** TUNEL assay of apoptotic VSMCs (green). **(D)** JC-1 staining. TCM treatment reduced mitochondrial membrane potential (green). Bars, 100 μm.

To verify our speculation, the apoptotic activity of VSMCs was detected by three different approaches. VSMCs were stained with Annexin V-FITC/PI, and analyzed by flow cytometry. The cell apoptosis rate of TCM-treated group (33.8±2.26%) was higher than that of the control (17.22±1.36%) (P<0.01) (Figure 3A). Moreover, TCM-induced apoptosis at both early and advanced stages was also significantly increased compared with control (*P*<0.01) (Figure 3A). Meantime, immunofluorescence staining also showed the similar results. Annexin V-positive and PI-positive cells in VSMCs treated with TCM were more than that of the control group (Figure 3B). The similar results were found by TdT-mediated dUTP nick end labeling (TUNEL) assay, which displayed increased TUNEL-positive nuclei in TCM-treated cells (Figure 3C). Additionally, we also tested the effect of TCM on the mitochondrial membrane potentials in VSMCs. Cells were labeled with JC1 dye, and measured by fluorescence microscope. TCM treatment resulted in loss of mitochondrial membrane potential in VSMCs (Figure 3D). These results suggest that TCM induces cell death via mitochondrial apoptotic pathway in VSMCs.

### TCM induces the activation of Caspase-3 in VSMCs

**Figure 4:**
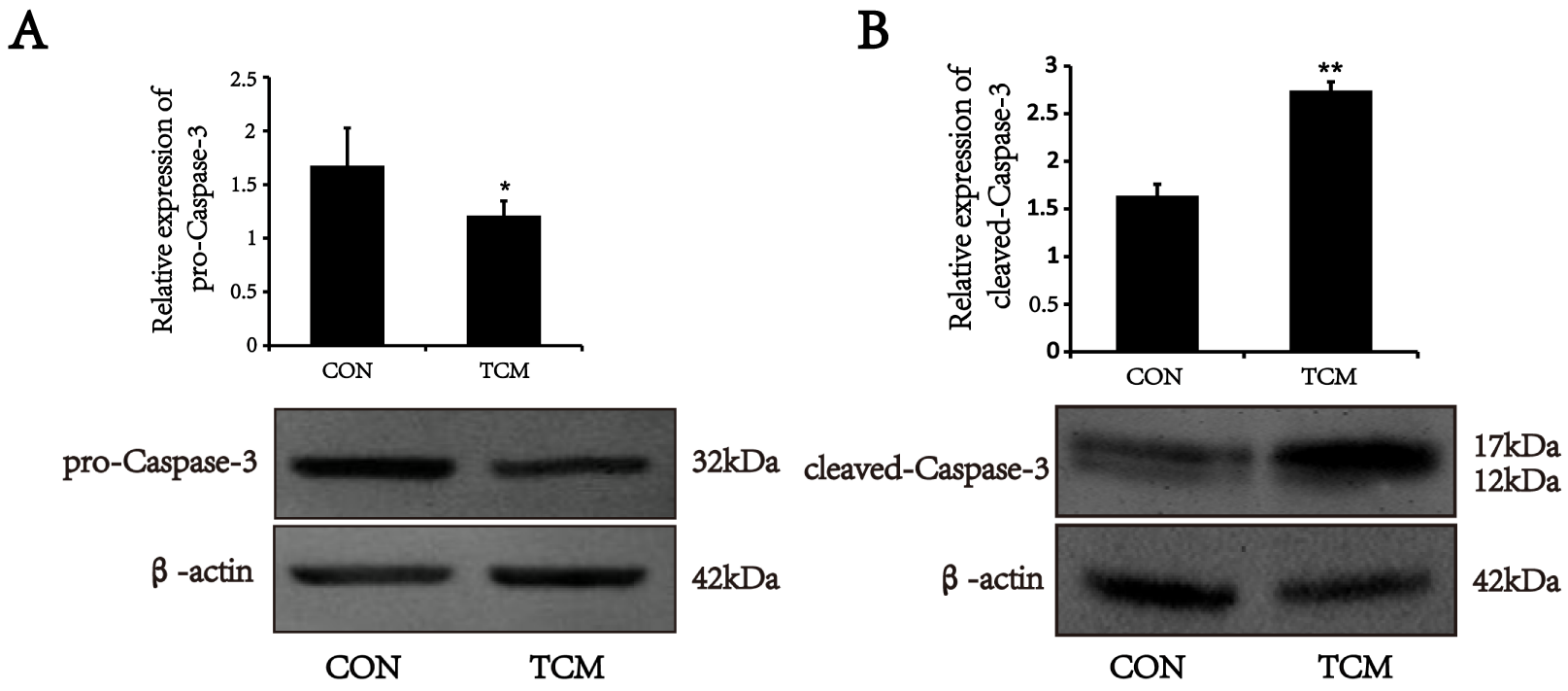
Effects of TCM on the activation of caspase-3 in VSMCs **(A)** Western blot analysis of pro-caspase-3 in VSMCs. **(B)** Western blot analysis of cleaved-caspase-3 in VSMCs. Bar graphs show the means ± SEM from three independent experiments (n=3). *P < 0.05, **P < 0.01 versus the CON.

Caspase-3 activation is a critical determinant of mitochondria apoptosis pathway [9]. To test whether the apoptosis of VSMCs induced by TCM is associated with Caspase cascades, Caspase-3 expression was analyzed by Western blot. The results showed that pro-Caspase-3 protein level markedly reduced in VSMCs treated by TCM (*P*<0.05) (Figure 4A). However, the expression of cleaved-Caspase-3 increased under the same conditions (*P*<0.01) (Figure 4B). The results indicate that the caspase cascades are initiated in the apoptosis induced by TCM.

### TCM increases the expression of bax, and inhibits the expression of Bcl-2 in VSMCs

**Figure 5:**
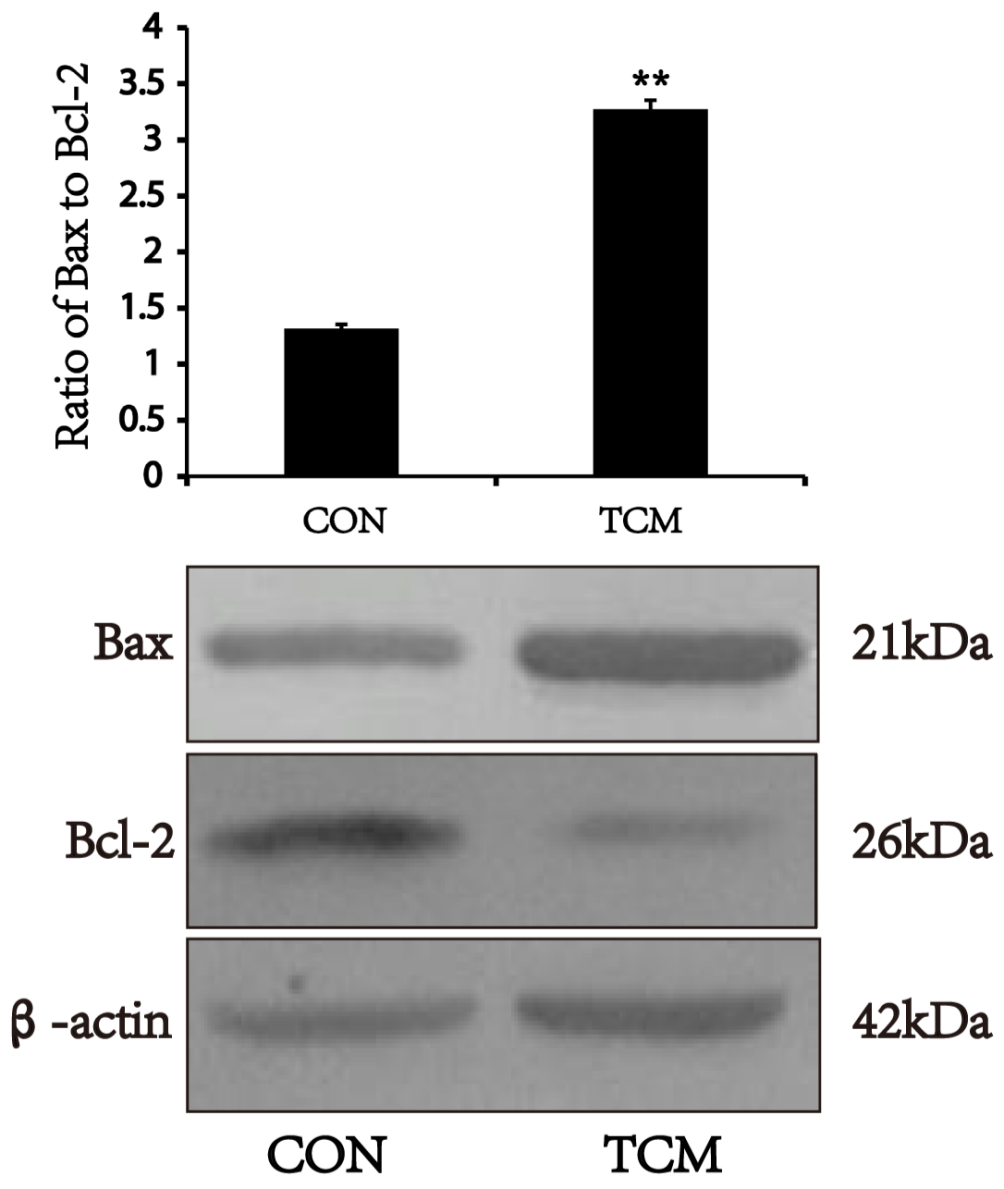
Effects of TCM on the expression of Bax and Bcl-2 in VSMCs Western blot analysis of Bax and Bcl-2 in VSMCs. Bar graphs show the means ± SEM from three independent experiments (n=3). **P < 0.01 versus the CON.

Bcl-2 and Bax are the most representative antiapoptotic and proapoptotic genes of Bcl-2 family [10]. We examined whether TCM modulate the expression of Bcl-2 and Bax by Western blot, and showed that the expression of Bcl-2 protein reduced in VSMCs after TCM treatment. Both Bax expression and the ratio of Bax to Bcl-2 increased under the same conditions (*P*<0.01) (Figure 5). The results suggest that TCM may induce apoptosis of VSMCs through the mitochondrial pathway.

## DISCUSSION

The growth of the solid tumors beyond 2–3 mm in greatest dimension depends on the development of tumor angiogenesis [11]. Most tumor vessel walls showed structural abnormalities, such as the absence of endothelial cells and lack of smooth muscle layer [8]. Although the mechanism of tumor vascular structure abnormalities have been studied in-depth, the pathological mechanism of the lack of smooth muscle layer of tumor blood vessels is still lack of sufficient attention and exploration. In the present study, we used TCM to mimic the tumor microenvironment *in vitro* and demonstrated the exocrine active factors from the CRC cells induced apoptosis of VSMCs through a mitochondrial caspase pathway.

We showed that the level of SMA expression in the tumor vessels in colorectal cancer was significantly lower than that in matched normal colorectal tissues which displayed a constant high density of smooth muscle cells. The colorectal cancer vessels lacked full muscular coat in comparison with that of normal colorectal tissues. Furthermore, TCM reduced the viability of VSMCs in a concentration-dependent manner, but not change the expression of PCNA, a marker of cell proliferation***.*** Therefore, we speculated that the decreased cell number may result from apoptosis. The defective walls of tumor vessels break the rules of normal blood-vessel construction. This may aid metastasis by facilitating the movement of tumor cells into the bloodstream.

Apoptosis is an evolutionarily conserved suicide process that plays a critical role in embryonic development and in the homeostasis, remodeling, surveillance, and host defenses of postnatal tissues [12]. One of the major apoptosis pathways is mitochondrial pathway. The most critical mitochondrial events during apoptosis are the structural and functional remodeling of this organelle and subsequent release of apoptogenic proteins into the cytosol. In the present study, TCM incubation induced VSMC apoptosis at both early and advanced stages, accompanied with loss of mitochondrial membrane potential. This suggested that some active components in TCM may activate mitochondrial apoptotic pathway.

Caspases are central to the execution of apoptosis. Pro-caspases-3, which exist as preformed inactive dimers, are activated by proteolytic cleavage. The cleaved-Caspase-3 performs the proteolytic destruction of the cell [13]. We showed that pro-Caspase-3 protein level was down-regulated in VSMCs treated by TCM, while cleaved-Caspase-3 was increased under the same conditions. It is clear that in mitochondrial apoptotic pathway Bcl-2 and Bax are one of the most important antiapoptotic and proapoptotic proteins respectively [14]. TCM treatment resulted in reduced expression of Bcl-2 and increased Bax in VSMCs, indicating that the caspase cascades mediates the apoptosis of VSMC induced by TCM. As glucose stress can induce the cell apoptosis, in the present study we did not exclude the conditions such as glucose starvation of the medium after 24-hour incubation with the cell line.

In conclusion, our findings suggest that the TCM from the tumor cells induces VSMC apoptosis through the mitochondrial pathway. Caspase cascades may mediate tumor matrix-induced VSMC apoptosis, contributing to the heterogeneity of tumor vasculature. The analysis of the active components secreted from the tumor cells is performed in the future to explore the biomarkers targeting vascular cells.

## MATERIALS AND METHODS

### Tissue preparation

Approval was obtained from the Hebei Medical University Clinical Research Ethics Committee. After informed consent had been given, the tumor and matched adjacent normal tissues, which were over 10 cm from tumor edges, were obtained from CRC treated with surgery alone at Hebei Medical University Forth Hospital, China between October 2014 and November 2015. The samples were snap frozen in liquid nitrogen within a maximum of 30 min after their removal, and stored at −80°C and were fixed in formalin for detection by immunohistochemistry.

### Animals

Sprague Dawley rats were obtained from the Experimental Animal Center of Hebei Medical University. Only male mice, at least 12 weeks of age, 80–100 g, were used for experiments. All animal procedures were conducted in accordance with the Guide for the Care and Use of Laboratory Animals published by the US National Institutes of Health (NIH Publication, 8th Edition, 2011), and were approved by the Institutional Animal Care and Use Committee of Hebei Medical University.

### Cell culture and treatment

Sprague-Dawley rats were sacrificed, the aorta was removed. VSMCs were isolated as described previously [15]. VSMCs were cultured in Dulbecco’s modified Eagle’s medium (DMEM, GIBCO) supplemented with 10% fetal bovine serum(FBS, GIBCO) and maintained under 5% CO2 at 37 °C in a humidified atmosphere. The cells from passages 3–6 were used in all experiments. HT-29 cells[ATCC HTB-38] are a colonic epithelial line that was obtained from Cell Bank of the Chinese Academy of Sciences (Shanghai, China).

TCM from HT-29 cells cultured in McCoy’s 5A containing 10% FBS was collected, and filtered through a 0.22-μm filter, and used to treat VSMCs. McCoy’s 5A was used as a control medium.

### Cell counting and MTT assay

Cell numbers were determined using a hemocytometer as previously described [16]. Each count was an average of five repeats, and each data point represents an average of three experiments. After appropriate treatment, the viability of VSMCs was measured using the MTT assay as previous described [17].

### Western blot analysis

The harvested cells were washed and homogenized on ice with RIPA buffer (50 mM TRis–Cl, pH 7.5, 150 mM NaCl, 1% NP-40, 0.5% sodium deoxycholate and 1 mM phenylmethanesulfonyl fluoride). The lysates containing equal amounts of protein were separated by SDS-PAGE, and electroblotted onto polyvinylidene difluoride membranes by a semi-dry transfer system (Bio-Rad). Blots were blocked with 5% nonfat dry milk in TBST (10 mM Tris–Cl, pH 7.5, 150 mM NaCl, 0.1% Tween-20) for 2 h at room temperature, then probed with mouse monoclonal antibody against β-actin (1:800, Santa Cruz), mouse polyclonal antibody against Bcl-2(1:800, ABclonal), rabbit polyclonal antibody against Bax (1:1000, ABclonal), rabbit polyclonal antibody against PCNA (1:1000, Santa Cruz) and rabbit polyclonal antibody against Caspase-3 (1:800, Cell Signaling Technology) at 4°C overnight, then washed with TBST and incubated with fluorescence-conjugated goat antimouse/rabbit IgG (Rockland) for 1 h at room temperature. The immunoreactive bands were visualized on Odyssey Infrared Imaging System (Li-Cor Biosciences), and the protein expression was quantified by densitometric analysis using Odyssey Imaging Software v3.0 (Li-Cor Biosciences). The experiment was repeated three times.

### Immunohistochemistry analysis

Sections were blocked with 0.3% hydrogen peroxide, followed by preincubation with 5% normal goat serum and then incubation with primary antibodies against CD34 (1:100, Abcam) and SMA (1:100, Abcam)at 4°C overnight. Next, the sections were incubated with the biotinylated secondary antibody, followed by streptavidin-horseradish peroxidase and diaminobenzidine, and then counterstained with hematoxylin. Staining intensities of SMA were determined by measurement of the mean optical density (MOD) by light microscopy using a computer-based Image-Pro Plus System. CD34 Staining was determined by MVD assessment. MVD was assessed using the Chalkley grid method, whereby tissue sections were examined at low magnification (40×) and five areas of increased MVD (vascular hotspots) were identified as previously described [18].

### Cell apoptosis assay

Cell apoptosis was measured by flow cytometry and fluorescence microscope, respectively. For flow cytometry, briefly, after treatment, cells were collected and stained with Annexin V and PI staining using annexin V-FITC apoptosis kit (YEASEN, Shanghai, China) according to the manufacturer’s instruction. Early apoptotic (annexin V-positive and PI-negative) cells were distinguished from late apoptotic (annexin V and PI double-positive) or necrotic (PI-positive) cells by a flow cytometric analysis. The assay of Annexin V-FITC/PI, mitochondrial membrane potentials (JC-1) (Beyotime, Guangzhou, China) and TUNEL (qcbio, Shanghai, China) was carried out according to the manufacturer’s protocol.

### Statistical analysis

Data analysis was performed using SPSS version 13.0. Data are presented as means±SEM. Quantitative values of protein expressions in CRC and the matched normal tissues were analyzed with paired t-tests. Other paired data were compared using Student’s t-test. Differences between groups were determined with one-way analysis of variance (ANOVA) with Dunnett-T test. A probability value of < 0.05 was considered significant.

## Abbreviations

CRC: colorectal cancer
VSMC: vascular smooth muscle cell
TCM: tumor conditioned medium
SMA: smooth muscle α-actin
PCNA: proliferating cell nuclear antigen
VEGF: vascular endothelial growth factor
FGF: fibroblast growth factor
PDGF: platelet derived growth factor
EGF: epidermal growth factor
MVD: microvascular density
TUNEL: TdT-mediated dUTP nick end labeling
MOD: mean optical density
